# Verrucous form of Hansen disease: a diagnostic dilemma

**DOI:** 10.1093/skinhd/vzaf114

**Published:** 2026-01-28

**Authors:** Prajwal Pudasaini, Sushil Paudel, Sagar GC, Bandana Parajuli, Prashanta Pudasaini, Esna Thapa, Faisal Dubash

**Affiliations:** Department of Dermatology, Civil Service Hospital, Minbhawan, Kathmandu, Bagmati Provence, Nepal; Department of Dermatology, Civil Service Hospital, Minbhawan, Kathmandu, Bagmati Provence, Nepal; Department of Dermatology, Civil Service Hospital, Minbhawan, Kathmandu, Bagmati Provence, Nepal; Department of Dermatology, Civil Service Hospital, Minbhawan, Kathmandu, Bagmati Provence, Nepal; Department of Surgery, Helping Hands Community Hospital, Kathmandu, Bagmati Provence, Nepal; Department of Internal Medicine, Sahid Gangalal National Heart Centre, Kathmandu, Bagmati Provence, Nepal; Department of Dermatology, Ninewells Hospital and Medical School, Dundee, UK

## Abstract

In a leprosy endemic country like Nepal, Hansen disease should always be kept in the list of differential diagnoses due to its varied clinical features and also it being a common mimicker.

Dear Editor, A 23-year-old man presented with a gradually progressive, asymptomatic, rough, elevated plaque of 9 months’ duration over the extensor aspect of his right elbow, wrist and knees. The plaque started as a pea-sized papule and gradually increased in size over a period of 3 months with enhanced verrucosity of the surface (Figures 1, 2). He was previously treated with topical salicylic acid 40% ointment along with cryotherapy at another medical centre for a suspected diagnosis of verruca vulgaris. However, the lesions persisted despite this treatment. There was no history of numbness of the affected area, or neurological signs or symptoms. On examination, rough hyperkeratotic plaque with whitish overlying scales and crust was present over the right elbow, wrist and right knee. The largest plaque of the elbow region measured 2 × 1 cm over the right elbow, along with evolving plaque over the medial part on the proximal aspect of hand. Biopsy was performed to confirm the diagnosis, which showed acanthosis and pseudoepethiliomatous hyperplasia of the epidermis. The dermis showed aggregates of foamy macrophages involving periadnexal and perivascular structures. Wade–Fite stain was positive for lepra bacilli and the bacillary index was 4 (Figures 3–5). The most likely diagnosis was the verrucous form of Hansen disease. The other probable differential diagnoses were atypical mycobacterial infection, chromoblastomycosis, verruca vulgaris and callosities.

**Figure 1:**
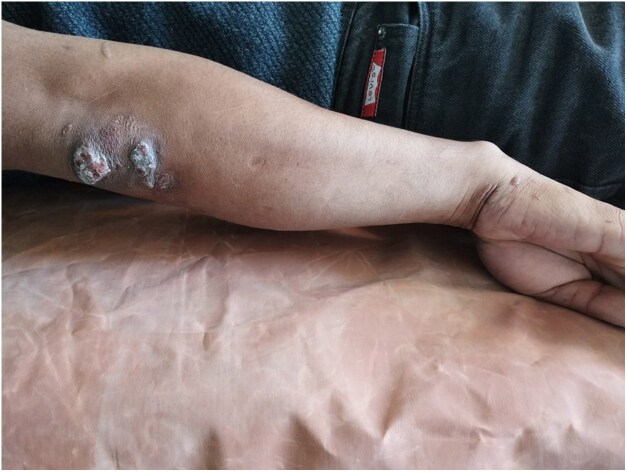
Hyperkeratotic plaque over the right elbow region.

**Figure 2:**
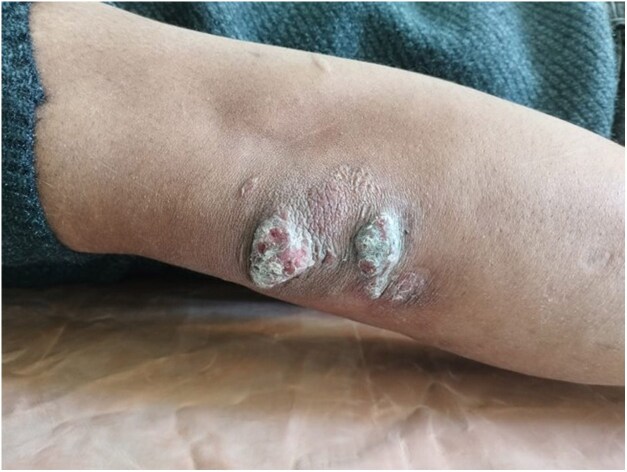
Close-up view of the same lesion of extensor aspect of the right elbow.

**Figure 3:**
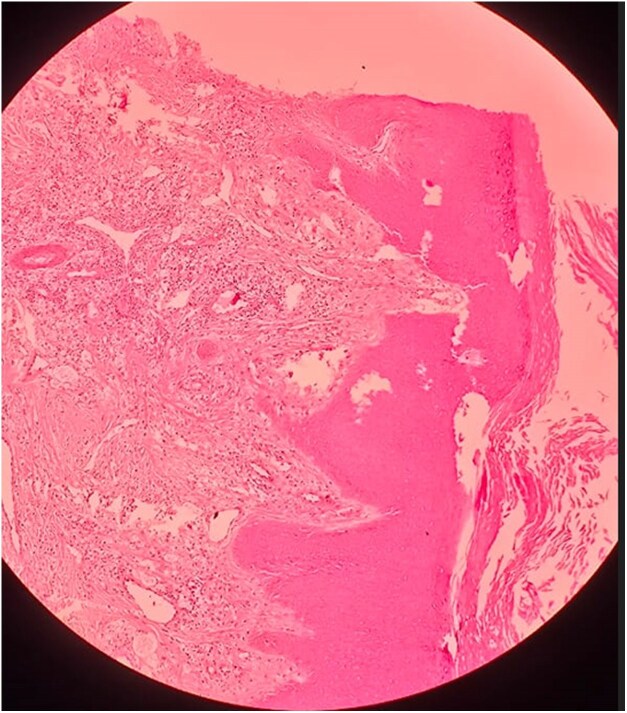
Acanthosis and pseudoepethiliomatous hyperplasia of the epidermis. Dermis shows aggregates of foamy macrophage involving periadnexal and perivascular structure. Haematoxylin and eosin stain (×4 magnification).

**Figure 4:**
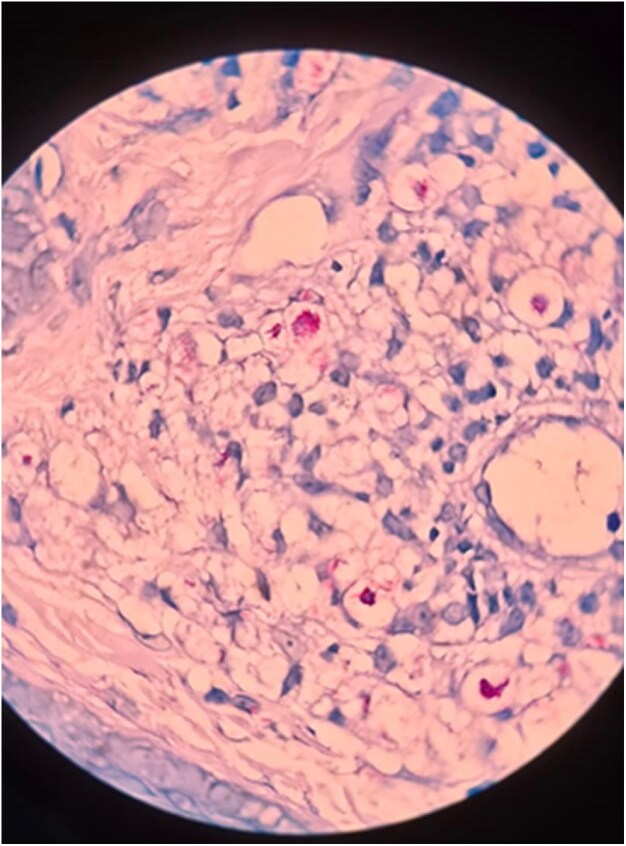
Dermis showed aggregates of foamy macrophage involving periadnexal and perivascular structure. Wade–Fite stain was positive for lepra bacilli. Haematoxylin and eosin stain (×10 magnification).

**Figure 5:**
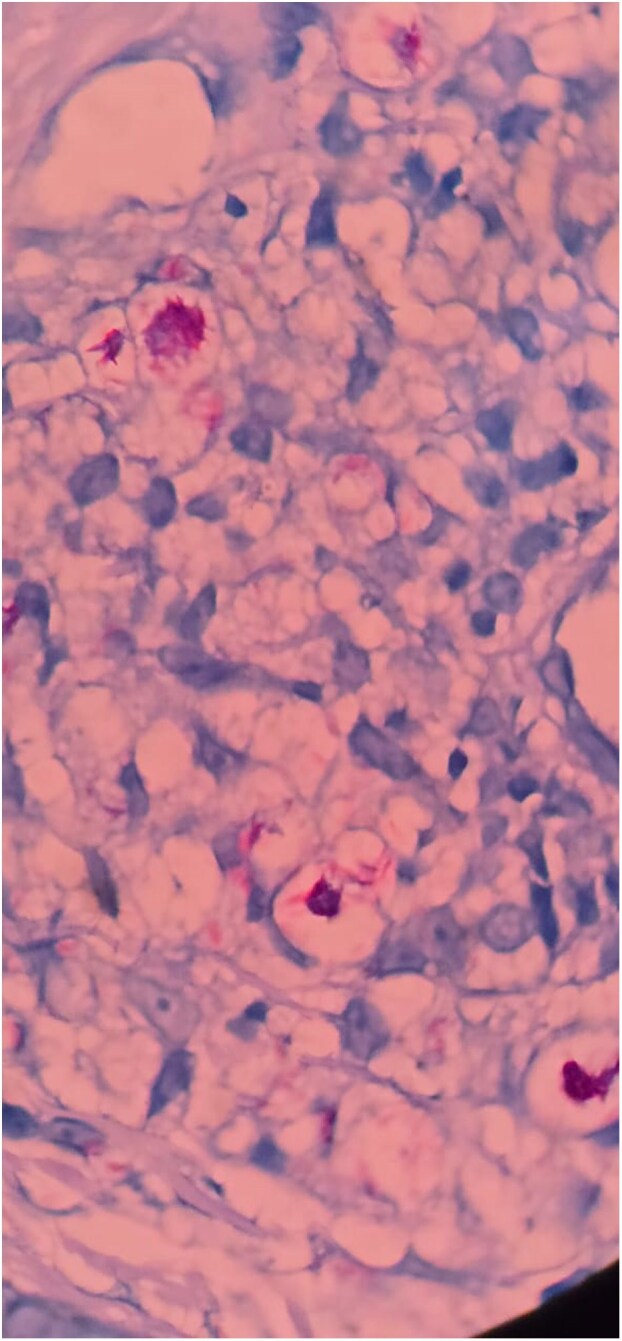
Positive Wade–Fite stain for lepra bacilli with a bacillary index of 4. Haematoxylin and eosin stain (×40 magnification).

Hansen disease is a chronic granulomatous infection caused by an intracellular acid-fast bacterium – *Mycobacterium leprae*. It commonly affects skin, nerves and the eyes, and in the most severe form there is involvement of various body systems and organs, including joints, kidneys, liver, testes and ovaries.^1^ Based on immunological response to lepra bacilli, it is further subclassified into a spectrum of diseases along with the two extreme subtypes: the tuberculoid and lepromatous forms. Among the various subtypes of lepromatous leprosy, the verrucous form is a rarer subtype and is often difficult to diagnose due to its clinical resemblance to other ubiquitous verrucous lesions such as verruca vulgaris.^2^ Given the multisystemic nature of this chronic mycobacterial disease, prompt diagnosis and treatment are prudent to preventing disseminated disease with neural damage, disability and deformities, which can cause significant social stigma in underserved regions. In a leprosy endemic country like Nepal, Hansen disease should always be kept on the list of differential diagnoses due to its varied clinical features and being a common mimicker.

## Data Availability

All data in this study are included in this published article.
